# A Fatal Case of Acute Suppurative Otitis

**Published:** 1880-08

**Authors:** F. C. Hotz

**Affiliations:** Chicago


### Clinical Reports.

### NOTES FROM PRIVATE PRACTICE.

### ARTICLE V.

*A Fatal Case of Acute Suppurative Otitis.*

It happens but very seldom that an acute inflammation of the middle ear causes the death of the patient in the first week or fortnight. For this reason I thought that the report of the following case, which I have recently observed in consultation with Dr. S. D. Jacobson of this city, would not be without interest. The doctor has kindly furnished me the following memorandum of his observations:

April 23, 1880. Was called to see Mrs. S. M., 56 years old, of full habit, but enjoying good health until three or four days ago, when she was seized with violent pain in her head, particularly in the right side; also some deafness and a humming noise in the right ear. The next day there was already a purulent discharge from the ear, which, however, brought no relief. I found the patient up and dressed, with her head wrapped in a woolen shawl; face flushed, pulse 96, temp. 38.50, tongue coated; complained of intense headache, earache, loss of appetite and of sleep, constipation; could not hear the watch tick with right ear unless it was in contact with skull; with left ear heard watch at six inches. No complaints on left side of head. On the right side great tenderness of ear and mastoid region, purulent discharge from meatus. Ordered hot-water injections in ear, an aperient and iodide of potassium with bromide of potassium internally.

April 26. Improving. Headache not so much. Discharge diminishing, as also tenderness of right ear. Commenced to-day to complain of severe pains in left ear, with tinnitus in the same; watch not heard except when in contact. No discharge from left ear. Tympanum almost normal.

April 28. Improving slowly; had less pain.

April 29. Had a chill to-day, followed by heat and perspiration; temperature 40.2 in the evening. Ordered quinine.

April 30, morning. Temperature 39; slept after morph. syrup; headache intense; no discharge; membrana tympani of a dull, red appearance not bulging. On account of intense pain: paracentesis.

May 1. Purulent discharge from left ear since yesterday; headache less; had another chill with perspiration afterwards.

In the forenoon of May 2, I saw the patient and noted the following condition: Temperature 40, pulse 80; tongue coated and dry; eyes half closed; pupils of equal size; patient did not answer to questions, indifferent to everything, though conscious; but very restless, constantly moving the upper and lower extremities. Right ear, moderate purulent discharge, without any complication. Left ear, a small quantity of sanguinolent pus in external meatus; large opening in membrana tympani, showing the mucous membrane of the tympanic cavity in a highly vascular and swollen state. There was some swelling and induration behind, above and in front of the ear; it was more marked in the temporal region than any where else.

In the afternoon of the same day, Dr. J. found complete paralysis of left side of face and right side of body; loss of consciousness; retention of urine, and involuntary evacuation of bowels; temperature 41. In this comatose state the patient continued thirty-six hours, and died early in the morning of May 4th.

I regret that the autopsy could not be obtained in this case; for it would have been very interesting to ascertain positively the extent and nature of the fatal complication, and also to find out—if it was possible—why the disease took that insidious course in the left ear, while it proved so mild and manageable in the right ear. Without autopsy our diagnosis amounts only to a conjecture suggested by the prominent symptoms of the disease. The succession of the graver symptoms was as follows: Violent inflammation of the left tympanum, repeated chills, œdema about the ear, cerebral excitation followed by coma and paralysis. This train of symptoms, I think warrants the conclusion to be drawn that death resulted probably from thrombosis and phlebitis of the lateral sinus, and pyæmia. As evidence of thrombosis and phlebitis, I regard the œdema about the ear and the cerebral excitation preceding the comatose stage ; while the repeated chills and high fever are manifestations of pyæmia.

One symptom which is considered pretty good evidence of phlebitis of the lateral sinus, but which was not clearly developed in the above case, is an induration and swelling of the tissues of the neck. The presence of this symptom in one case, and its absence in another case, is easily explained. We know that the lateral sinus is in direct communication with the cervical veins through the *venæ emissariæ mastoideæ*, which emerge just behind the mastoid process. These emissary veins therefore furnish an easy route by which the phlebitis of the lateral sinus can be propagated to the extra-cranial veins. Whenever this occurs, we shall probably observe the induration and swelling in the tissues of the neck, which, together with the other symptoms, will aid us materially in diagnosticating the intra-cranial phlebitis. But the phlebitis need not necessarily take this course ; the emissary veins need not be implicated, and then, of course, the above symptom will not be observed.

Dr. J. Orne Green has recently published* an interesting account of four cases in which the diagnosis of phlebitis of the lateral sinus was chiefly based upon this induration and swelling in the tissues of the neck. All of the cases were fatal, and in the fourth case the autopsy was obtained and confirmed the diagnosis. In two cases there had been a discharge from the ear for many years, and the fatal phlebitis was developed in the course of an acute relapse of the suppurative inflammation ; in two cases it followed an acute inflammation of the tympanum.

A very typical case of this kind was reported by me at the meeting of the Illinois State Medical Society in 1876.† The case took an unusually rapid course and sudden end ; and as the

---

* *American Journal of Otology*, July, 1879, and April, 1880.

† Transact. of Ill. State Med. Soc. 1876, p. 89.

transactions of the State society are accessible but to a very limited number, I will reproduce it.

"*Acute Suppurative Otitis; Phlebitis and Thrombosis of the Lateral Sinus; Death from Pyœmia.*

"Chas. L., forty-five years old, had always been in good health, except suffering from chronic catarrh of the naso-pharyngeal space and from a slight deafness. In February, 1876, he had an attack of acute catarrhal otitis, and a large syphilitic ulcer in the left tonsil. Both affections were speedily cured; but after taking cold again, he returned, on February 28th, complaining of great pain in the right ear. External ear and mastoid region showed nothing abnormal; meatus was wide and natural; membrana tympani intensely red. The weather being very unsettled, I advised the patient to remain in the house, and ordered leeches behind the ear, and hot water poultices.

"March 1.—Leeches gave great relief; but instead of warm water, dry compresses were put on the ear. During last night he had violent pain; no appetite; pulse 90; tongue coated; meatus so swollen that membrana tympani could not be seen. Mastoid region neither red nor swollen, but about the mastoid foramen there was a small spot which was exceedingly tender. Leeches again.

"March 2.—After the leeching the pain subsided, and the ear began to discharge. Still he was very restless last night. Meatus less swollen, filled with muco-purulent secretions; membrana tympani perforated. Morphia at eight.

"March 3.—Slept well and felt very much better, tongue cleaner, appetite improving; no pain in the ear or head; that tenderness about the mastoid foramen, and great restlessness his only complaint. He was up all day, took a light dinner and supper and retired about 9 o'clock feeling quite well. After midnight he complained of feeling chilly though the room had a very comfortable temperature; very soon after this he had a violent chill of half an hour, followed by high fever, quickened respiration and delirium. Towards morning he vomited several times and became comatose.

"March 4.—I found him in the following state: Pulse 120 and small; skin hot and covered with profuse clammy perspiration; face sallow; eyes widely opening and pupils dilated; did not recognize anybody, and was talking continuously. The discharge from the ear which was very copious last night had a chocolate color. No swelling of the mastoid region; but below it there was a diffuse, indurated swelling of the tissues and a redness of the integument of the neck; the induration followed chiefly the course of the internal jugular vein and was evidently very tender, for when I pressed on it the patient groaned. Respiration very quick, and superficial auscultation did not reveal anything abnormal. In this comatose state the patient continued until his death which occured in the afternoon.

"*Autopsy.*—Eighteen hours after death. The right side of the neck showed extensive redness of the skin and a diffuse swelling of the tissues. No œdema of mastoid region; but its subcutaneous tissues extensively reddened by hæmorrhagic effusion due to the leeching; periosteum and bone normal. Skull of normal thickness; sub-arachnoidean spaces contained some serum; pia mater hazy over both hemispheres; the veins filled by a very dark liquid blood. Substance of brain firm, its ventricles empty.

"The left lateral sinus in its entire length was filled with dark, liquid blood. In the right lateral sinus, as far as it runs along the petrous portion of the temporal bone, we found a greyish red thrombus which was firmly adherent to one side of the sinus and which could be traced down into the jugular foramen. The jugular vein was filled by a dense firm clot which presented the brownish granular appearance of a coagulum formed some time before death; the inner coat of the vein was red and slightly rough—indicating the existence of phlebitis. A section of the common carotid, several inches long, was removed, but it presented nothing abnormal. The petrous bone was red, but not softened or eroded. The mucous membrane of the tympanic cavity was intensely red; the cavity as well as the mastoid cells were filled with puriform matter."

Dr. Danforth submitted the brain to further macroscopic and microscopic investigations, and kindly furnished me with the following notes: "The right cerebral lobe was intensely congested in every part; the meningeal vessels were generally occluded by clots. The minute vessels of the pia mater and the more superficial vessels of the brain substance were very generally filled with a granular matter. This embolic condition was the rule in all the minute vessels of both hemispheres of the cerebrum."

In this case the different phases of the disease, from the simple acute inflammation of the tympanic cavity to the pyæmia, succeeded each other very rapidly, and the autopsy revealed the succession to have been as follows: acute suppurative otitis, involving early the mastoid cells; extension of the inflammation to the lateral sinus, which is separated from the cells merely by a thin osseous wall, perforated usually by numerous foramina, for the passage of minute veins from the cells to the sinus; phlebitis of the sinus and formation of a thrombus which extended into the jugular vein; pieces of this thrombus were torn off, broken up into small detritus while circulating with the blood, and lodged as numerous emboli in the smallest arterioles of the brain and meninges, causing instant coma and death.

F. C. HOTZ, M.D.

CHICAGO.

